# Methyl (2*Z*)-2-bromo­meth­yl-3-(3-chloro­phen­yl)prop-2-enoate

**DOI:** 10.1107/S1600536813012117

**Published:** 2013-05-11

**Authors:** K. Swaminathan, K. Sethusankar, Raman Selvakumar, Manickam Bakthadoss

**Affiliations:** a Department of Physics, RKM Vivekananda College (Autonomous), Chennai 600 004, India; b Department of Organic Chemistry, University of Madras, Maraimalai Campus, Chennai 600 025, India

## Abstract

There are two independent mol­ecules (*A* and *B*) in the asymmetric unit of the title compound C_11_H_10_BrClO_2_, which represents the *Z* isomer. The methyl­acrylate moieties are essentially planar, within 0.084 (2) and 0.027 (5) Å in mol­ecules *A* and *B*, respectively. The benzene ring makes dihedral angles of 13.17 (7) and 27.89 (9)° with the methyl­acrylate moiety in mol­ecules *A* and *B*, respectively. The methyl­bromide moiety is almost orthogonal to the benzene ring, making dihedral angles of 81.46 (16)° in mol­ecule *A* and 79.61 (16)° in mol­ecule *B*. The methyl­acrylate moiety exhibits an extended *trans* conformation in both mol­ecules. In the crystal, pairs of C—H⋯O hydrogen bonds result in the formation of quasi-centrosymmetric *R*_2_^2^(14) *AB* dimers.

## Related literature

For the uses of cinnamic acid and its derivatives, see: De *et al.* (2011 ▶); Sharma (2011 ▶). For an extended acrylate conformation, see: Schweizer & Dunitz (1982 ▶). For a related structure, see: Swaminathan *et al.* (2013 ▶). For graph-set notation, see: Bernstein *et al.* (1995 ▶)
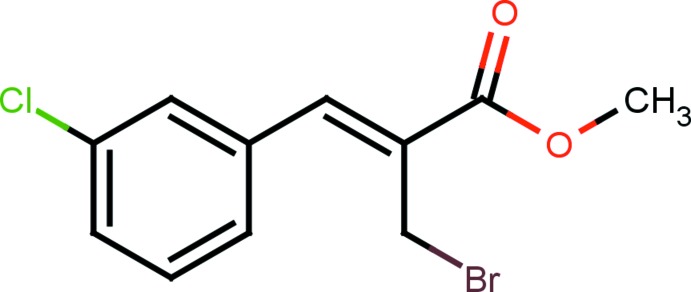


## Experimental

### 

#### Crystal data

C_11_H_10_BrClO_2_
*M* *_r_* = 289.54
Triclinic, 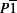

*a* = 7.4523 (3) Å
*b* = 11.7003 (4) Å
*c* = 14.3121 (5) Å
α = 72.078 (2)°
β = 76.539 (2)°
γ = 76.773 (2)°
*V* = 1137.98 (7) Å^3^
*Z* = 4
Mo *K*α radiation
μ = 3.82 mm^−1^
*T* = 296 K
0.30 × 0.25 × 0.20 mm

#### Data collection

Bruker Kappa APEXII CCD diffractometer
Absorption correction: multi-scan (*SADABS*; Bruker, 2008 ▶) *T*_min_ = 0.330, *T*_max_ = 0.466
27124 measured reflections
6597 independent reflections
4205 reflections with *I* > 2σ(*I*)
*R*_int_ = 0.032

#### Refinement

*R*[*F* ^2^ > 2σ(*F* ^2^)] = 0.036
*wR*(*F* ^2^) = 0.092
*S* = 1.00
6597 reflections
273 parameters
H-atom parameters constrained
Δρ_max_ = 0.85 e Å^−3^
Δρ_min_ = −0.49 e Å^−3^

### 

Data collection: *APEX2* (Bruker, 2008 ▶); cell refinement: *SAINT* (Bruker, 2008 ▶); data reduction: *SAINT*; program(s) used to solve structure: *SHELXS97* (Sheldrick, 2008 ▶); program(s) used to refine structure: *SHELXL97* (Sheldrick, 2008 ▶); molecular graphics: *ORTEP-3 for Windows* (Farrugia, 1997 ▶); software used to prepare material for publication: *SHELXL97* and *PLATON* (Spek, 2009 ▶).

## Supplementary Material

Crystal structure: contains datablock(s) global, I. DOI: 10.1107/S1600536813012117/ld2100sup1.cif

Structure factors: contains datablock(s) I. DOI: 10.1107/S1600536813012117/ld2100Isup2.hkl

Supplementary material file. DOI: 10.1107/S1600536813012117/ld2100Isup3.cml

Additional supplementary materials:  crystallographic information; 3D view; checkCIF report

## Figures and Tables

**Table 1:** Hydrogen-bond geometry (Å, °)

*D*—H⋯*A*	*D*—H	H⋯*A*	*D*⋯*A*	*D*—H⋯*A*
C1*A*—H1*A*⋯O1*B* ^i^	0.93	2.53	3.429 (3)	161
C1*B*—H1*B*⋯O1*A* ^i^	0.93	2.51	3.380 (3)	156

Symmetry code: (i) 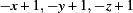.

**Fig. 1.**
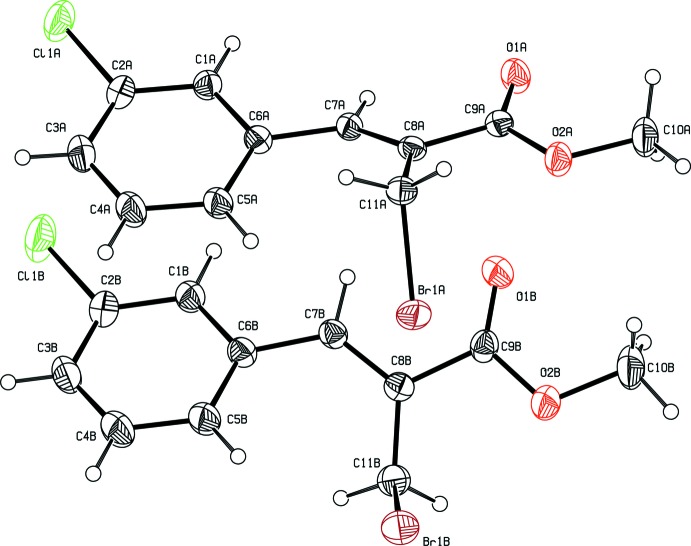
The molecular structure of the title compound with the atom numbering scheme. Displacement ellipsoids are drawn at 30% probability level. H atoms are present as small spheres of arbitary radius.

**Fig. 2.**
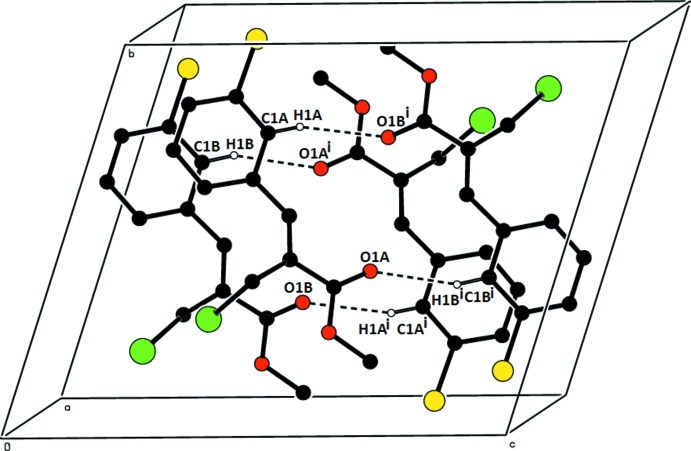
The crystal structure of the title compound, showing the formation of quasi-centrosymmetric R22(14) dimers. Hydrogen bonds are shown as dotted lines. The hydrogen atoms not involved in bonding have been omitted for the sake of clarity.

## supplementary crystallographic information

### Comment

Cinnamic acid derivatives are naturally occurring substances found in fruits, vegetables, flowers *etc.* and are consumed as dietary phenolic compounds. Different substitutions on basic moiety lead to various pharmacological activities like antioxidant, hepatoprotective, anxiolytic, insect repellent, antidiabetic, anticholesterolemic *etc*. (Sharma, 2011). Cinnamic acid derivatives received much attention in medicinal research as traditional as well as recent synthetic antitumor agents. (De *et al.*, 2011).

X-ray analysis established the molecular structure and atom connectivity of the title compound C_11_H_10_BrClO_2_, as illustrated in Fig. 1. The title compound comprises two crystallographically independent molecules in the asymmetric unit. The corresponding bond lengths and bond angles of both the molecules agree well with each other.

The methylacrylate moiety is essentially planar with a maximum deviation of 0.0843 (23) Å for atom C7A in the molecule A and 0.0271 (50) Å for atom C10B in the molecule B. Also the least square planes of the methylacrylate moiety form dihedral angles of 13.17 (7)° and 27.89 (9)°, with the least square planes of the respective benzene rings, in the molecules A and B, respectively.

The methylacrylate moieties adopt an extended conformation, as evident from the torsion angle values: [C7A–C8A–C9A–O1A = 11.0 (3)°, C7A–C8A–C9A–O2A = -170.21 (19)°, C8A–C9A–O2A–C10A = -179.1 (2)° and O1A–C9A–O2A–C10A = -0.3 (3)°] for the molecule A and [C7B–C8B–C9B–O1B = -2.9 (3)°, C7B–C8B–C9B–O2B = 177.7 (2)°, C8B–C9B–O2B–C10B = -179.1 (2)° and O1B–C9B–O2B–C10B = 0.7 (4)°] for the molecule B. The reasons for the extended conformation were discussed earlier (Schweizer and Dunitz, 1982).

In the molecule A, the phenyl ring and the carbonyl group of the acrylate are (+)*syn*-periplanar to each other with the torsion angle of C7A–C8A–C9A–O1A = 11.0 (3)° whereas in the molecule B, they are (-)*syn*-periplanar to each other with the torsion angle of C7B–C8B–C9B–O1B = -2.9 (3)°. Likewise, the carbonyl group of the acrylate and the methylbromide group are (-)anti-periplanar to each other with the torsion angle of C11A–C8A–C9A–O1A = -165.5 (2)°, in the molecule A while they are (+)anti-periplanar to each other with the torsion angle of C11B–C8B–C9B–O1B = 172.4 (2)°, in the molecule B.

The least square plane of methylbromide group in the molecule A, forms dihedral angles of 81.46 (16) and 85.04 (13)° with the phenyl ring and the acrylate group, respectively, being almost orthogonal to both. Similarly, the least square plane of methyl bromide group in the molecule B, forms dihedral angles of 79.61 (16) and 81.51 (16)° with the phenyl ring and the acrylate group, respectively, being nearly orthogonal to both. The title compound exhibits structural similarities with a related structure reported earlier (Swaminathan *et al.* 2013).

The crystal packing is stabilized by intermolecular C1A—H1A···O1B^i^ and C1B—H1B···O1A^i^ hydrogen bonds which form quasi-centrosymmetric *R*_2_^2^(14) dimers. The symmetry code: (i) -*x* + 1,-*y* + 1,-*z* + 1. The packing view of the title compound is shown in Fig.2.

### Experimental

To a stirred solution of methyl 2-((3-chlorophenyl)(hydroxy)methyl) acrylate (4 mmol) in CH_2_Cl_2_ (15 ml), 48% aqueous HBr (0.68 ml) was added at room temperature. The reaction mixture was cooled to 273 K and then catalytic amount of concentrated H_2_SO_4_ was added dropwise. The reaction mixture was stirred well at room temperature for about 24 hrs. After the completion of the reaction (confirmed by TLC analysis), the reaction mixture was poured into water and the aqueous layer was extracted with ethyl acetate (3 *x* 10 ml). The combined organic layer was washed with brine (10 ml) and concentrated. The crude product thus obtained was purified by column chromatography (EtOAc/Hexane, 2–6%) to provide Methyl (2*Z*)-2-(bromomethyl)-3-(3-chlorophenyl)prop-2-enoate in 90% yield, as a yellow crystalline solid.

### Refinement

Hydrogen atoms were placed in calculated positions with C—H = 0.93 - 0.97 Å and refined in riding model with fixed isotropic displacement parameters: *U*_iso_(H) = 1.2 *U*_eq_(C) for aromatic and methylene groups *U*_iso_(H) = 1.5 *U*_eq_(O) for methyl group. The rotation angles for methyl group were optimized by least squares.

### Crystal data

**Table:**

C_11_H_10_BrClO_2_	*Z* = 4
*M**_r_* = 289.54	*F*(000) = 576
Triclinic, *P*1	*D*_x_ = 1.690 Mg m^−^^3^
Hall symbol: -P 1	Mo *K*α radiation, λ = 0.71073 Å
*a* = 7.4523 (3) Å	Cell parameters from 4205 reflections
*b* = 11.7003 (4) Å	θ = 2.7–30.0°
*c* = 14.3121 (5) Å	µ = 3.82 mm^−^^1^
α = 72.078 (2)°	*T* = 296 K
β = 76.539 (2)°	Block, colourless
γ = 76.773 (2)°	0.30 × 0.25 × 0.20 mm
*V* = 1137.98 (7) Å^3^	

### Data collection

**Table:**

Bruker Kappa APEXII CCD diffractometer	6597 independent reflections
Radiation source: fine-focus sealed tube	4205 reflections with *I* > 2σ(*I*)
Graphite monochromator	*R*_int_ = 0.032
ω & φ scans	θ_max_ = 30.0°, θ_min_ = 2.7°
Absorption correction: multi-scan (*SADABS*; Bruker, 2008)	*h* = −10→8
*T*_min_ = 0.330, *T*_max_ = 0.466	*k* = −16→16
27124 measured reflections	*l* = −20→19

### Refinement

**Table:**

Refinement on *F*^2^	Primary atom site location: structure-invariant direct methods
Least-squares matrix: full	Secondary atom site location: difference Fourier map
*R*[*F*^2^ > 2σ(*F*^2^)] = 0.036	Hydrogen site location: inferred from neighbouring sites
*wR*(*F*^2^) = 0.092	H-atom parameters constrained
*S* = 1.00	*w* = 1/[σ^2^(*F*_o_^2^) + (0.0394*P*)^2^ + 0.4208*P*] where *P* = (*F*_o_^2^ + 2*F*_c_^2^)/3
6597 reflections	(Δ/σ)_max_ < 0.001
273 parameters	Δρ_max_ = 0.85 e Å^−^^3^
0 restraints	Δρ_min_ = −0.49 e Å^−^^3^

### Special details

**Table:**

Geometry. All e.s.d.'s (except the e.s.d. in the dihedral angle between two l.s. planes) are estimated using the full covariance matrix. The cell e.s.d.'s are taken into account individually in the estimation of e.s.d.'s in distances, angles and torsion angles; correlations between e.s.d.'s in cell parameters are only used when they are defined by crystal symmetry. An approximate (isotropic) treatment of cell e.s.d.'s is used for estimating e.s.d.'s involving l.s. planes.
Refinement. Refinement of *F*^2^ against ALL reflections. The weighted *R*-factor *wR* and goodness of fit *S* are based on *F*^2^, conventional *R*-factors *R* are based on *F*, with *F* set to zero for negative *F*^2^. The threshold expression of *F*^2^ > σ(*F*^2^) is used only for calculating *R*-factors(gt) *etc*. and is not relevant to the choice of reflections for refinement. *R*-factors based on *F*^2^ are statistically about twice as large as those based on *F*, and *R*- factors based on ALL data will be even larger.

### Fractional atomic coordinates and isotropic or equivalent isotropic displacement parameters (Å^2^)

**Table:**

	*x*	*y*	*z*	*U*_iso_*/*U*_eq_	
C1A	0.2876 (3)	0.7438 (2)	0.31195 (17)	0.0375 (5)	
H1A	0.3090	0.7571	0.3690	0.045*	
C1B	0.7397 (3)	0.6245 (2)	0.15677 (18)	0.0427 (5)	
H1B	0.7468	0.6413	0.2151	0.051*	
C2A	0.2785 (3)	0.8381 (2)	0.22676 (18)	0.0427 (5)	
C2B	0.7449 (3)	0.7153 (2)	0.06871 (19)	0.0471 (6)	
C3A	0.2509 (4)	0.8220 (3)	0.14019 (19)	0.0520 (6)	
H3A	0.2441	0.8869	0.0832	0.062*	
C3B	0.7299 (4)	0.6945 (3)	−0.0185 (2)	0.0535 (7)	
H3B	0.7325	0.7568	−0.0775	0.064*	
C4A	0.2337 (4)	0.7075 (3)	0.13990 (19)	0.0535 (7)	
H4A	0.2181	0.6947	0.0815	0.064*	
C4B	0.7111 (4)	0.5795 (3)	−0.0165 (2)	0.0622 (8)	
H4B	0.7003	0.5643	−0.0748	0.075*	
C5A	0.2392 (4)	0.6116 (2)	0.22469 (17)	0.0449 (6)	
H5A	0.2258	0.5351	0.2232	0.054*	
C5B	0.7082 (4)	0.4864 (3)	0.07061 (18)	0.0515 (6)	
H5B	0.6956	0.4092	0.0705	0.062*	
C6A	0.2651 (3)	0.6286 (2)	0.31333 (16)	0.0349 (5)	
C6B	0.7240 (3)	0.5078 (2)	0.15876 (17)	0.0387 (5)	
C7A	0.2722 (3)	0.5341 (2)	0.40719 (15)	0.0332 (5)	
H7A	0.3350	0.5491	0.4501	0.040*	
C7B	0.7119 (3)	0.4161 (2)	0.25545 (17)	0.0362 (5)	
H7B	0.6619	0.4469	0.3105	0.043*	
C8A	0.2045 (3)	0.42983 (19)	0.44251 (15)	0.0322 (5)	
C8B	0.7620 (3)	0.2947 (2)	0.27668 (17)	0.0357 (5)	
C9A	0.2376 (3)	0.3555 (2)	0.54369 (16)	0.0345 (5)	
C9B	0.7259 (3)	0.2279 (2)	0.38432 (18)	0.0400 (5)	
C10A	0.2170 (4)	0.1673 (3)	0.6645 (2)	0.0570 (7)	
H10A	0.3460	0.1550	0.6707	0.085*	
H10B	0.1820	0.0900	0.6734	0.085*	
H10C	0.1399	0.2051	0.7143	0.085*	
C10B	0.7412 (6)	0.0366 (3)	0.5018 (2)	0.0936 (13)	
H10D	0.8176	0.0562	0.5383	0.140*	
H10E	0.7731	−0.0486	0.5051	0.140*	
H10F	0.6116	0.0554	0.5303	0.140*	
C11A	0.0906 (3)	0.3861 (2)	0.39206 (17)	0.0383 (5)	
H11A	0.0254	0.4555	0.3480	0.046*	
H11B	−0.0026	0.3441	0.4417	0.046*	
C11B	0.8615 (3)	0.2251 (2)	0.20373 (19)	0.0462 (6)	
H11C	0.9573	0.1618	0.2332	0.055*	
H11D	0.9236	0.2794	0.1455	0.055*	
O1A	0.2956 (3)	0.38962 (15)	0.60031 (12)	0.0480 (4)	
O1B	0.6636 (3)	0.27411 (17)	0.45134 (13)	0.0552 (5)	
O2A	0.1916 (2)	0.24515 (15)	0.56628 (12)	0.0459 (4)	
O2B	0.7732 (3)	0.10769 (17)	0.39833 (14)	0.0643 (5)	
Cl1A	0.30217 (12)	0.98211 (6)	0.22889 (6)	0.0646 (2)	
Cl1B	0.76375 (14)	0.86058 (7)	0.06837 (6)	0.0776 (2)	
Br1A	0.24789 (4)	0.27505 (2)	0.314378 (19)	0.04929 (9)	
Br1B	0.69480 (5)	0.14955 (3)	0.16130 (2)	0.06111 (10)	

### Atomic displacement parameters (Å^2^)

**Table:**

	*U*^11^	*U*^22^	*U*^33^	*U*^12^	*U*^13^	*U*^23^
C1A	0.0424 (12)	0.0350 (13)	0.0363 (12)	−0.0070 (9)	−0.0090 (10)	−0.0092 (10)
C1B	0.0519 (14)	0.0411 (14)	0.0362 (13)	−0.0080 (11)	−0.0114 (10)	−0.0091 (11)
C2A	0.0468 (13)	0.0323 (13)	0.0462 (14)	−0.0061 (10)	−0.0112 (11)	−0.0048 (11)
C2B	0.0503 (14)	0.0406 (14)	0.0472 (15)	−0.0100 (11)	−0.0118 (11)	−0.0034 (12)
C3A	0.0648 (17)	0.0471 (16)	0.0375 (14)	−0.0092 (13)	−0.0129 (12)	0.0006 (12)
C3B	0.0583 (16)	0.0582 (18)	0.0349 (14)	−0.0101 (13)	−0.0069 (11)	−0.0004 (12)
C4A	0.0760 (18)	0.0533 (17)	0.0336 (13)	−0.0131 (14)	−0.0172 (12)	−0.0079 (12)
C4B	0.089 (2)	0.064 (2)	0.0360 (15)	−0.0127 (16)	−0.0175 (14)	−0.0123 (14)
C5A	0.0637 (15)	0.0401 (14)	0.0341 (13)	−0.0130 (12)	−0.0100 (11)	−0.0104 (11)
C5B	0.0742 (18)	0.0477 (16)	0.0380 (14)	−0.0128 (13)	−0.0145 (12)	−0.0139 (12)
C6A	0.0366 (11)	0.0365 (13)	0.0316 (11)	−0.0061 (9)	−0.0060 (9)	−0.0092 (10)
C6B	0.0420 (12)	0.0416 (14)	0.0335 (12)	−0.0084 (10)	−0.0078 (9)	−0.0097 (10)
C7A	0.0395 (11)	0.0313 (12)	0.0298 (11)	−0.0065 (9)	−0.0075 (9)	−0.0082 (9)
C7B	0.0410 (12)	0.0378 (13)	0.0323 (12)	−0.0108 (10)	−0.0070 (9)	−0.0096 (10)
C8A	0.0348 (11)	0.0355 (12)	0.0285 (11)	−0.0031 (9)	−0.0063 (9)	−0.0133 (10)
C8B	0.0356 (11)	0.0383 (13)	0.0366 (12)	−0.0089 (9)	−0.0074 (9)	−0.0123 (10)
C9A	0.0384 (11)	0.0318 (12)	0.0336 (12)	−0.0063 (9)	−0.0038 (9)	−0.0109 (10)
C9B	0.0486 (13)	0.0337 (13)	0.0403 (13)	−0.0108 (10)	−0.0143 (10)	−0.0064 (11)
C10A	0.0814 (19)	0.0398 (15)	0.0432 (15)	−0.0159 (14)	−0.0144 (14)	0.0049 (12)
C10B	0.176 (4)	0.0428 (18)	0.0502 (19)	−0.021 (2)	−0.019 (2)	0.0061 (15)
C11A	0.0401 (12)	0.0402 (13)	0.0383 (12)	−0.0075 (10)	−0.0080 (10)	−0.0145 (10)
C11B	0.0472 (13)	0.0444 (15)	0.0469 (14)	−0.0084 (11)	−0.0044 (11)	−0.0144 (12)
O1A	0.0712 (11)	0.0431 (10)	0.0357 (9)	−0.0166 (8)	−0.0183 (8)	−0.0075 (8)
O1B	0.0819 (13)	0.0480 (11)	0.0358 (9)	−0.0105 (9)	−0.0100 (9)	−0.0121 (8)
O2A	0.0687 (11)	0.0326 (9)	0.0382 (9)	−0.0164 (8)	−0.0133 (8)	−0.0037 (7)
O2B	0.1103 (17)	0.0354 (11)	0.0442 (11)	−0.0108 (10)	−0.0138 (10)	−0.0073 (9)
Cl1A	0.0915 (5)	0.0330 (4)	0.0676 (5)	−0.0149 (3)	−0.0219 (4)	−0.0024 (3)
Cl1B	0.1185 (7)	0.0414 (4)	0.0735 (5)	−0.0251 (4)	−0.0339 (5)	0.0048 (4)
Br1A	0.06512 (17)	0.04343 (16)	0.04765 (16)	−0.00739 (12)	−0.01453 (12)	−0.02202 (12)
Br1B	0.0890 (2)	0.05188 (18)	0.05424 (18)	−0.01782 (15)	−0.01588 (15)	−0.02436 (14)

### Geometric parameters (Å, º)

**Table:**

C1A—C2A	1.373 (3)	C7A—H7A	0.9300
C1A—C6A	1.389 (3)	C7B—C8B	1.340 (3)
C1A—H1A	0.9300	C7B—H7B	0.9300
C1B—C2B	1.375 (3)	C8A—C11A	1.488 (3)
C1B—C6B	1.388 (3)	C8A—C9A	1.486 (3)
C1B—H1B	0.9300	C8B—C11B	1.480 (3)
C2A—C3A	1.376 (3)	C8B—C9B	1.489 (3)
C2A—Cl1A	1.743 (2)	C9A—O1A	1.202 (3)
C2B—C3B	1.376 (4)	C9A—O2A	1.335 (3)
C2B—Cl1B	1.736 (3)	C9B—O1B	1.194 (3)
C3A—C4A	1.375 (4)	C9B—O2B	1.333 (3)
C3A—H3A	0.9300	C10A—O2A	1.448 (3)
C3B—C4B	1.375 (4)	C10A—H10A	0.9600
C3B—H3B	0.9300	C10A—H10B	0.9600
C4A—C5A	1.378 (3)	C10A—H10C	0.9600
C4A—H4A	0.9300	C10B—O2B	1.452 (4)
C4B—C5B	1.380 (4)	C10B—H10D	0.9600
C4B—H4B	0.9300	C10B—H10E	0.9600
C5A—C6A	1.405 (3)	C10B—H10F	0.9600
C5A—H5A	0.9300	C11A—Br1A	1.971 (2)
C5B—C6B	1.395 (3)	C11A—H11A	0.9700
C5B—H5B	0.9300	C11A—H11B	0.9700
C6A—C7A	1.457 (3)	C11B—Br1B	1.969 (2)
C6B—C7B	1.465 (3)	C11B—H11C	0.9700
C7A—C8A	1.335 (3)	C11B—H11D	0.9700
			
C2A—C1A—C6A	120.3 (2)	C8B—C7B—H7B	115.1
C2A—C1A—H1A	119.9	C6B—C7B—H7B	115.1
C6A—C1A—H1A	119.9	C7A—C8A—C11A	125.72 (19)
C2B—C1B—C6B	120.1 (2)	C7A—C8A—C9A	116.34 (18)
C2B—C1B—H1B	119.9	C11A—C8A—C9A	117.84 (19)
C6B—C1B—H1B	119.9	C7B—C8B—C11B	125.4 (2)
C1A—C2A—C3A	121.8 (2)	C7B—C8B—C9B	115.7 (2)
C1A—C2A—Cl1A	118.87 (18)	C11B—C8B—C9B	118.7 (2)
C3A—C2A—Cl1A	119.3 (2)	O1A—C9A—O2A	123.0 (2)
C1B—C2B—C3B	121.5 (2)	O1A—C9A—C8A	125.0 (2)
C1B—C2B—Cl1B	119.2 (2)	O2A—C9A—C8A	112.00 (18)
C3B—C2B—Cl1B	119.3 (2)	O1B—C9B—O2B	122.9 (2)
C4A—C3A—C2A	118.4 (2)	O1B—C9B—C8B	125.4 (2)
C4A—C3A—H3A	120.8	O2B—C9B—C8B	111.7 (2)
C2A—C3A—H3A	120.8	O2A—C10A—H10A	109.5
C4B—C3B—C2B	118.5 (3)	O2A—C10A—H10B	109.5
C4B—C3B—H3B	120.7	H10A—C10A—H10B	109.5
C2B—C3B—H3B	120.7	O2A—C10A—H10C	109.5
C3A—C4A—C5A	121.1 (2)	H10A—C10A—H10C	109.5
C3A—C4A—H4A	119.5	H10B—C10A—H10C	109.5
C5A—C4A—H4A	119.5	O2B—C10B—H10D	109.5
C3B—C4B—C5B	121.0 (2)	O2B—C10B—H10E	109.5
C3B—C4B—H4B	119.5	H10D—C10B—H10E	109.5
C5B—C4B—H4B	119.5	O2B—C10B—H10F	109.5
C4A—C5A—C6A	120.3 (2)	H10D—C10B—H10F	109.5
C4A—C5A—H5A	119.8	H10E—C10B—H10F	109.5
C6A—C5A—H5A	119.8	C8A—C11A—Br1A	111.48 (15)
C4B—C5B—C6B	120.2 (3)	C8A—C11A—H11A	109.3
C4B—C5B—H5B	119.9	Br1A—C11A—H11A	109.3
C6B—C5B—H5B	119.9	C8A—C11A—H11B	109.3
C1A—C6A—C5A	118.0 (2)	Br1A—C11A—H11B	109.3
C1A—C6A—C7A	116.98 (19)	H11A—C11A—H11B	108.0
C5A—C6A—C7A	125.0 (2)	C8B—C11B—Br1B	113.16 (16)
C1B—C6B—C5B	118.5 (2)	C8B—C11B—H11C	108.9
C1B—C6B—C7B	117.6 (2)	Br1B—C11B—H11C	108.9
C5B—C6B—C7B	123.7 (2)	C8B—C11B—H11D	108.9
C8A—C7A—C6A	131.42 (19)	Br1B—C11B—H11D	108.9
C8A—C7A—H7A	114.3	H11C—C11B—H11D	107.8
C6A—C7A—H7A	114.3	C9A—O2A—C10A	115.59 (19)
C8B—C7B—C6B	129.8 (2)	C9B—O2B—C10B	114.8 (2)
			
C6A—C1A—C2A—C3A	−1.3 (4)	C1B—C6B—C7B—C8B	−153.1 (2)
C6A—C1A—C2A—Cl1A	178.57 (18)	C5B—C6B—C7B—C8B	31.3 (4)
C6B—C1B—C2B—C3B	1.7 (4)	C6A—C7A—C8A—C11A	−3.2 (4)
C6B—C1B—C2B—Cl1B	179.77 (19)	C6A—C7A—C8A—C9A	−179.4 (2)
C1A—C2A—C3A—C4A	−0.5 (4)	C6B—C7B—C8B—C11B	6.4 (4)
Cl1A—C2A—C3A—C4A	179.6 (2)	C6B—C7B—C8B—C9B	−178.6 (2)
C1B—C2B—C3B—C4B	−0.6 (4)	C7A—C8A—C9A—O1A	11.0 (3)
Cl1B—C2B—C3B—C4B	−178.7 (2)	C11A—C8A—C9A—O1A	−165.5 (2)
C2A—C3A—C4A—C5A	1.6 (4)	C7A—C8A—C9A—O2A	−170.21 (19)
C2B—C3B—C4B—C5B	−0.3 (4)	C11A—C8A—C9A—O2A	13.3 (3)
C3A—C4A—C5A—C6A	−0.8 (4)	C7B—C8B—C9B—O1B	−2.9 (3)
C3B—C4B—C5B—C6B	0.1 (5)	C11B—C8B—C9B—O1B	172.4 (2)
C2A—C1A—C6A—C5A	2.0 (3)	C7B—C8B—C9B—O2B	177.7 (2)
C2A—C1A—C6A—C7A	−178.6 (2)	C11B—C8B—C9B—O2B	−7.0 (3)
C4A—C5A—C6A—C1A	−1.0 (4)	C7A—C8A—C11A—Br1A	95.1 (2)
C4A—C5A—C6A—C7A	179.6 (2)	C9A—C8A—C11A—Br1A	−88.7 (2)
C2B—C1B—C6B—C5B	−1.9 (4)	C7B—C8B—C11B—Br1B	−101.0 (2)
C2B—C1B—C6B—C7B	−177.7 (2)	C9B—C8B—C11B—Br1B	84.2 (2)
C4B—C5B—C6B—C1B	1.0 (4)	O1A—C9A—O2A—C10A	−0.3 (3)
C4B—C5B—C6B—C7B	176.5 (2)	C8A—C9A—O2A—C10A	−179.1 (2)
C1A—C6A—C7A—C8A	157.6 (2)	O1B—C9B—O2B—C10B	0.7 (4)
C5A—C6A—C7A—C8A	−23.1 (4)	C8B—C9B—O2B—C10B	−179.8 (3)

### Hydrogen-bond geometry (Å, º)

**Table:**

*D*—H···*A*	*D*—H	H···*A*	*D*···*A*	*D*—H···*A*
C1*A*—H1*A*···O1*B*^i^	0.93	2.53	3.429 (3)	161
C1*B*—H1*B*···O1*A*^i^	0.93	2.51	3.380 (3)	156

Symmetry code: (i) −*x*+1, −*y*+1, −*z*+1.
